# Novel Isatin-based activator of p53 transcriptional functions in tumor cells

**DOI:** 10.22099/mbrc.2019.34179.1419

**Published:** 2019-09

**Authors:** Regina Mirgayazova, Raniya Khadiullina, Rimma Mingaleeva, Vitaly Chasov, Marina Gomzikova, Ekaterina Garanina, Albert Rizvanov, Emil Bulatov

**Affiliations:** 1 Kazan Federal University, Kazan, Russian Federation; 2 Shemyakin-Ovchinnikov Institute of Bioorganic Chemistry, Russian Academy of Sciences, Moscow, Russian Federation

**Keywords:** p53, Transcriptional activity, Isatin-Schiff base, Metal complex, Tumor cells

## Abstract

Bioinorganic medicinal chemistry remains a hot field for research aimed at developing novel anti-cancer treatments. Discovery of metal complexes as potent antitumor chemotherapeutics such as cisplatin led to a significant shift of focus toward organometallic/ bioinorganic compounds containing transition metals and their chelates as novel scaffolds for drug discovery. In that way, transition metal complexes coordinated to essential biological scaffolds represent a highly promising class of compounds for design of novel target-specific therapeutics. Here, we report novel data on p53 activating Isatin-based Cu(II) complex exhibiting cytotoxic properties towards HCT116 and MCF7 tumor cell lines, as confirmed by cell viability assay and flow cytometry analysis of apoptosis. Furthermore, putative p53-mediated mechanism of action of this compound is supported by quantitative analysis of *TP53*, *MDM2* and *PUMA* genes expression, as well as luciferase-based p53 pathway activation assay. Multiplex immunoassay analysis of inflammatory markers revealed potential modulation of several cytokines and chemokines.

## INTRODUCTION

Transcription factor p53 plays a cornerstone role in regulation of cell cycle and is undoubtedly one of the most important tumor suppressors. In normal cells in the absence of cellular stress, the levels of p53 protein and its activity remain relatively low. However, stress conditions that directly or indirectly affect the genome integrity lead to p53 activation. This process happens through a signaling cascade that involves phosphorylation and other post-translational modifications that result in expression of p53 target genes responsible for cell cycle arrest, DNA repair or apoptosis.

Under physiological conditions, low cellular levels of p53 protein are maintained by E3 ubiquitin ligase MDM2 that ubiquitinates p53 for subsequent recognition and cleavage by 26S proteasome. Cellular stress leads to impaired protein-protein interaction between p53 and MDM2 as a result of phosphorylation of both proteins [1].

About 50% of all human tumors express wild-type p53, and in a large proportion of tumors p53 functions incorrectly due to disrupted regulation of protein itself or its signaling cascade. Importantly, p53 is functionally inactivated in most tumors due to factors that include mutations in *TP53* gene, mostly occurring in DNA-binding domain [2, 3], and overexpression of its negative regulator MDM2 and its homolog MDM4 (also known as MDMX) [4]. In many instances p53 activity can be restored using small molecule modulators – such as activators of p53 mutants or MDM2 inhibitors.

Inactivation of p53 as a result of missense mutations is considered one of the most common molecular mechanisms of p53 dysfunction [5]. Reactivation of p53 mutants by means of small molecules was previously reported for PRIMA-1 and its methylated analog APR-246 [6]. Oncogenic mutation Y220C represents a particularly lucrative molecular target for small molecule modulation due to availability of a well-defined druggable pocket [7]. Several examples of small molecule activators of p53(Y220C) mutant have been reported in the past, including PhiKan083 [8], L5 [9] and aminobenzothiazole MB725 [10], the most potent compound to date. 

The most prominent example of negative regulators of p53 includes ubiquitin ligases MDM2 and MDM4, members of the ubiquitin-proteasome system (UPS), responsible for ubiquitylation of p53 and its further degradation by 26S proteasome. The UPS components play a primary role in maintaining constant levels of various proteins in the cell, p53 in particular [11-13]. Activators of p53, including modulators of the UPS, represent a novel and highly promising class of compounds for therapeutic intervention in cancer and inflammatory diseases [14]. There are examples of metal complexes targeting MDM2-p53 protein-protein interaction and acting as therapeutic compounds for cancer treatment [15]. Interestingly enough, many of those molecules include Cu(II) complexes [16-18] and Isatin derivatives [19].

Isatin (1H-indole-2,3-dione) and its metabolites are components of multiple natural products produced by many plants, particularly belonging to genus *Isatis*. Isatin is also a metabolic derivative of adrenaline in humans [20]. There are reported FDA-approved tyrosine kinase inhibitors that share Isatin scaffold, i.e. Sunitinib for treatment of renal cell carcinoma, Nintedanib for idiopathic pulmonary fibrosis and non-small-cell lung cancer, and two experimental molecules that have failed Phase 3 clinical trials - Semaxanib for colorectal cancer and Orantinib for hepatocellular carcinoma.

Schiff bases are widely known for their role in coordination chemistry, these ligands are easy to synthesize, accessible, universal, capable of stabilizing metal ions in various oxidation states. Depending on nature of the starting materials (primary amines and carbonyl precursors) Schiff bases can exhibit variable denticity and functionality [21, 22]. These compounds used as ligands for complexation with transition metals, such as Co(II) [23] and Zn(II) and demonstrate bacteriostatic and carcinostatic activity upon chelation with metal ion [21, 22]. Schiff base derivatives of indole-2,3-dione (Isatin), demonstrated significant antiproliferative activity against various cancer cell lines, including human neuroblastoma SH-SY5Y [24-26], colorectal carcinoma HCT-116 [27] and melanoma TM1 cell lines [28].

Together with collaborators we have previously identified small molecule Isatin-Schiff base derivatives (ISBDs) that demonstrate p53-dependent anti-tumor activity  [29-32] . Here, we report the evaluation of cytotoxicity, transcriptional regulation and cytokine/chemokine profiling of this novel class of p53 activators in MCF7 breast adenocarcinoma and HCT116 colorectal carcinoma cell lines.

## MATERIALS AND METHODS

**Materials: **(E)-1-methyl-3-(phenylimino)indolin-2-one (Ligand) and (E)-1-methyl-3-(phenylimino)indolin-2-one copper(II) chloride complex (Complex) were obtained and characterized as previously reported [30, 32]. Dimethyl sulfoxide and CuCl_2_ were purchased from Sigma. Primers and Taqman probes were from Lytech (Russia).

**Cell culture: **MCF7 human breast adenocarcinoma and HCT116 human colorectal carcinoma cells were from American Type Culture Collection and grown on Dulbecco’s modified Eagle’s medium (PanEco, Russia). Cell media was supplemented with 10% fetal bovine serum (FBS, Biosera, France), penicillin/streptomycin and 2 mM L-glutamine. The cells were grown at 37°C in an atmosphere of 5% CO_2_ in air. 

**Treatments:** 5 mM stock solutions of Ligand and Complex were prepared just before the experiments by dissolving the lyophilized compounds in DMSO, whereas CuCl_2_ was dissolved in deionized water. Cell treatments were performed using Ligand and Complex at 50 μM, CuCl_2_ at 50 μM (1% DMSO) at 37°C in medium supplemented with serum. As negative control equal volumes of DMSO (1%) were added to untreated cells. Doxorubicin was used as a positive control at concentration of 4 μM.

**Immunoblotting analysis:** Cells were seeded in 6-well plates at 5×10^5^ cells per well and cultured for 2 days followed by treatment with Ligand (50 µM), Complex (50 µM), CuCl_2_ (50 µM), DMSO (1%) for 24 h. Treated cells were harvested and lysed in RIPA buffer (Thermo Fischer Scientific, USA) containing 1 μl/ml Halt Protease and Phosphatase Inhibitor Cocktail with EDTA (Thermo Fischer Scientific, USA) according to manufacturer’s protocol. Whole cell extracts were analysed using Pierce BCA Protein Assay Kit (Thermo Fischer Scientific, USA) to determine total protein concentration. Samples were fractioned by 10% SDS–PAGE and transferred to Immun-Blot PVDF membrane using Trans-Blot SD Semi-Dry Transfer Cell (Bio-Rad, USA). Membranes were blocked with PBS-T containing 5% (mass/vol) nonfat dried milk for 1 h at RT, incubated with primary anti-p53 antibodies (Abcam, USA) overnight at 4°C, and then with Anti-Mouse IgG–Peroxidase antibody (Sigma-Aldrich, USA) for 1 h. THE Beta Actin Antibody [HRP] (GenScript, USA) was used for detection of Beta Actin as loading control. Blots were developed with Clarity Western ECL Substrate (Bio-Rad, USA) and documented using ChemiDoc XRS Plus (Bio-Rad, USA). 

**p53 Luciferase reporter assay:** Cignal p53 Pathway Reporter Assay Kit (CCS-004L, SA Biosciences) was used to monitor the activity of the p53-regulated signal transduction pathway in MCF7 cells treated with Ligand, Complex, CuCl_2_, DMSO (vehicle control) following manufacturer’s instructions. Briefly, MCF7 cells were seeded at a density of 5×10^3^ per well in 96-well plates and grown to ~ 60% confluence. For each well, a mixture of inducible p53-responsive Firefly luciferase construct and constitutively expressing Renilla luciferase construct were co-transfected into the cells using Lipofectamine 3000 reagent (Invitrogen). 24 h after transfection cells were treated with Ligand (50 µM), Complex (50 µM), CuCl_2_ (50 µM), DMSO (1%) for 24 h. Firefly and Renilla luciferase activity measurements were carried out using Dual-Glo Luciferase Assay Kit (Promega) according to manufacturer’s protocol and Infinite M200 microplate reader (Tecan, Switzerland).

**MTS assay: **The cytotoxicity of Isatin Schiff base and its copper (II) complex were measured using standard colorimetric MTS assay. The cellular metabolic activities were assessed by the ability of their mitochondrial dehydrogenase to catalyze reduction of blue tetrazolium salt to colored formazan in a manner proportional to the number of viable cells. Cells were seeded into 96-well plates at 5×10^3^ cells per well in full DMEM medium for 24 h, after that DMSO (1%), Ligand (50 μM), Complex (50 μM), CuCl_2_ (50 μM) and doxorubicin 4 μM were added to appropriate wells and incubation continued for another 24 h. Then 20 μl of solution containing 2 mg/ml of MTS reagent (Promega, USA) and 150 μM phenazine methosulfate, PMS, (Dia-M, Russia) was added to each well for 3 h at 37°С. The absorbance at 490 nm was measured using Infinite M200 microplate reader (Tecan, Switzerland).

**Quantitative PCR: **The standard procedure of total RNA extraction was performed using TRIzol Reagent (Thermo Fisher Scientific, USA) according to the manufacturer’s instructions. Concentration of the extracted RNA was determined by optical density measurement (A260/A280 ratio) using NanoDrop 2000 Spectrophotometer (Thermo Fisher Scientific, USA). The reverse transcription reaction with 3 μg of total RNA was performed using 5x Reaction Buffer (Thermo Fisher Scientific, USA), RiboLock RNAse inhibitor (Thermo Fisher Scientific, USA), RevertAid (Thermo Fisher Scientific, USA), dNTP (Lytech, Russia) and Random 6 primer (Lytech, Russia). The reaction tubes were incubated at 25°C for 10 min, at 42°C for 60 min and at 70°C for 10 min using С1000 Thermal Cycler (Bio-Rad, CA, USA). Quantitative real-time PCR was carried out using 2.5x Master Mix (Sintol, Russia) and analyzed on the Bio-Rad CFX-96 real time system (Bio-Rad, CA, USA). Total volume of amplification reactions was 10 μL and each well was included 4 μL of 2.5x Master Mix, 1 μL of cDNA, 70-100 nM of both forward and reverse special primers. The PCR cycling conditions were following: 95°C for 3 min, 95°C for 10 sec, 55°C for 30 sec. The PCR was repeated for 49 cycles. Relative normalized expression was calculated by normalizing the cycle threshold values of genes of interest with those of β-actin, data analyzed in CFX Manager software. All PCR reactions were performed in triplicates.

Quantitative Taqman RT-PCR was performed using the following primers and probes. 

*TP53:* (fwd), (rev), [HEX] [BH2] (probe)

*MDM2*: TGT GCA AAG AAG CTA AAG AAA AGG (fwd), AGG TTG TCT AAA TTC CTA GGG TTA T (rev), [HEX] ATT GGT TGT CTA CAT ACT GGG CAG GG [BHQ2] (probe). *p21/CDKN1A*: GCC TCC TCA TCC CGT GTT CT (fwd), GTA CCA CCC AGC GGA CAA GT (rev), [HEX] AGC CGG CCC ACC CAA CCT CCG [BHQ2] (probe). *PUMA*: GGG CCC GTG AAG AGC AAA TG (fwd), CTG GCT CAG GGA AGA TGG CT (rev), [FAM] CGG TTG CTC CAG CCC GGC GC [BHQ1] (probe). *Beta Actin*: (fwd), (rev), [HEX] [BH2] (probe).

**Flow cytometry analysis: **Quantification of apoptotic cells was performed for MCF7 cells treated with Complex (50 µM), Ligand (50 µM), CuCl_2_ (50 μM) and DMSO (1%) for 48 h. Treated cells were harvested by trypsinization, washed with DPBS and stained using APC Annexin V Apoptosis Detection Kit with Propidium Iodide (Sony Biotechnology, USA) according to manufacturer’s protocol. Stained cells were immediately analyzed by flow cytometry using BD FACSAria III (BD Biosciences, USA) and data processed with FlowJo software package (FlowJo LLC, USA).

**Multiplex immunoassay: **Cells were seeded in 6-well plates at 5×10^5^ cells per well and cultured for 48 h followed by treatment with Ligand (50 µM), Complex (50 µM), CuCl_2_ (50 µM), DMSO (1%) for 24 h. Conditioned medium was collected from these cultures, centrifuged at 1500x g for 10 min, supernatants were collected and stored at -80°C. Bio-Plex Pro Human Cytokine 21-plex Assay kit (Bio-Rad) was used with Bio-Plex 200 system (Bio-Rad) for simultaneous qualitative and quantitative analysis of cytokines and chemokines. Supernatants were handled in accordance with the manufacturer's instructions. Concentrations (pg/ml) of analytes in test samples were determined using standard curves generated with eight concentration points. Nonlinear least squares minimization algorithm was used for curve fitting with five-parameter logistic equation to determine maximum and minimum treshold of detection. Data points above and below the detection range were discarded.

**Statistical analysis: **Statistical analysis was performed by two-tailed t-test in Origin 9 software package. Comparisons were considered statistically significant at p<0.05.

## RESULTS AND DISCUSSION

**Figure1:**
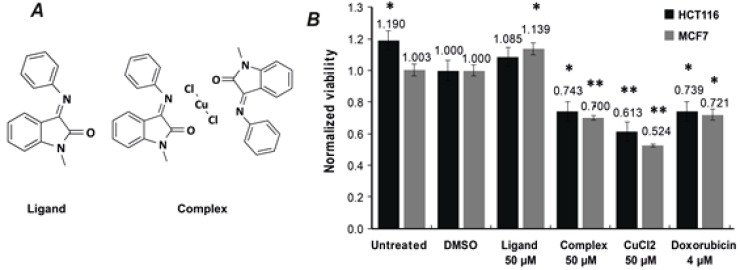
Isatin-Schiff base copper(II) complex negatively affects viability of HCT116 and MCF7 tumor cells.

One of the main objectives of the current study was to evaluate biological effect of Complex (Isatin-Schiff base copper(II) complex), our primary compound of interest, and Ligand (Isatin-Schiff base) towards two tumor cell lines, namely human colorectal cancer HCT116 and breast cancer MCF7. Chemical structures are shown in Fig. 1A. We compared the cytotoxicity of Complex with that of Ligand and CuCl_2_, each at 50 μM. Previously reported data shows that copper (II) complexes of Isatin-Schiff base derivatives negatively affect viability by inducing apoptosis in SH-SY5Y neuroblastoma cells [25, 26].

We performed cytotoxicity experiments using colorimetric MTS assay to determine viability of HCT116 and MCF7 tumor cell lines following 24 h treatment with the investigated compounds. Ligand demonstrated no cytotoxicity for either of the cell lines, whereas treatment with Complex and CuCl_2_ lead to substantially reduced viability of both cell lines that was comparable to doxorubicin (Fig. 1B). These results point out to two key findings: i) Ligand was significantly less toxic than Complex towards both cell lines, however once complexed with copper (II) its toxicity increased substantially; and ii) CuCl_2_ was more toxic for MCF7 than for HCT116 cells.

A – Chemical structures of (E)-1-methyl-3-(phenylimino)indolin-2-one (Ligand) and (E)-1-methyl-3-(phenylimino)indolin-2-one copper(II) chloride complex (Complex). B – HCT116 (black) and MCF7 (grey) cells were treated with Ligand (50 μM), Complex (50 μM), CuCl_2_ (50 μM) and doxorubicin 4 μM for 24 h. Cell viability was evaluated by colorimetric MTS assay. Ligand demonstrated no cytotoxicity for either of the cell lines, whereas treatment with Complex and CuCl_2_ lead to substantially reduced viability that was comparable to doxorubicin. Data are expressed as mean ± S.D., n = 3; *p<0.05, **p<0.001.

Transcription factor p53 plays a key function in triggering cell cycle arrest and apoptosis [33]. Previously reported ISBDs demonstrated p53-activating cellular effect, presumably occurring through MDM2 inhibition or/and DNA damage. Considering this presumptive mechanism of action and overexpression of MDM2 in breast cancer cell line MCF7 this line was chosen for immunoblot analysis.

**Figure 2:**
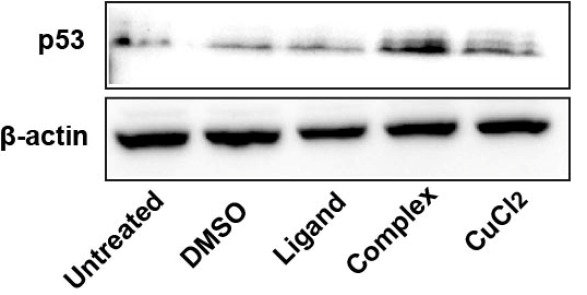
Treatment with Complex increases levels of p53 protein.

According to immunoblot analysis treatment of MCF7 cells with Complex enhanced p53 protein levels, whereas neither Ligand nor CuCl_2_ had any significant effect on p53 activation (Fig. 2). This leads to cautious assumption that by increasing p53 levels Complex can initiate p53-dependent signalling pathways. These results correlate with cytotoxicity data showing that Complex is more toxic than Ligand for both cell lines.

Next, we investigated effect of compounds on the expression levels of primary p53 target genes such as *MDM2 *(164785) and* PUMA *(605854), as well as *TP53 *(191170) gene itself. The p53-mediated transcription of *MDM2 *and* PUMA* genes leads to negative regulation of p53 activity by promoting its proteasomal degradation (MDM2) and induction of apoptosis via the mitochondrial pathway (PUMA) [34]. In our observations the compounds indeed altered the expression levels of these genes, although in a relatively modest manner.

Complex-mediated activation of p53 protein was confirmed by immunoblot analysis of MCF7 cells treated with Complex (50 μM), Ligand (50 μM), CuCl2 (50 μM) and DMSO (1%, vehicle control) for 24 h. Housekeeping protein β-actin was used as the loading control.

**Figure 3:**
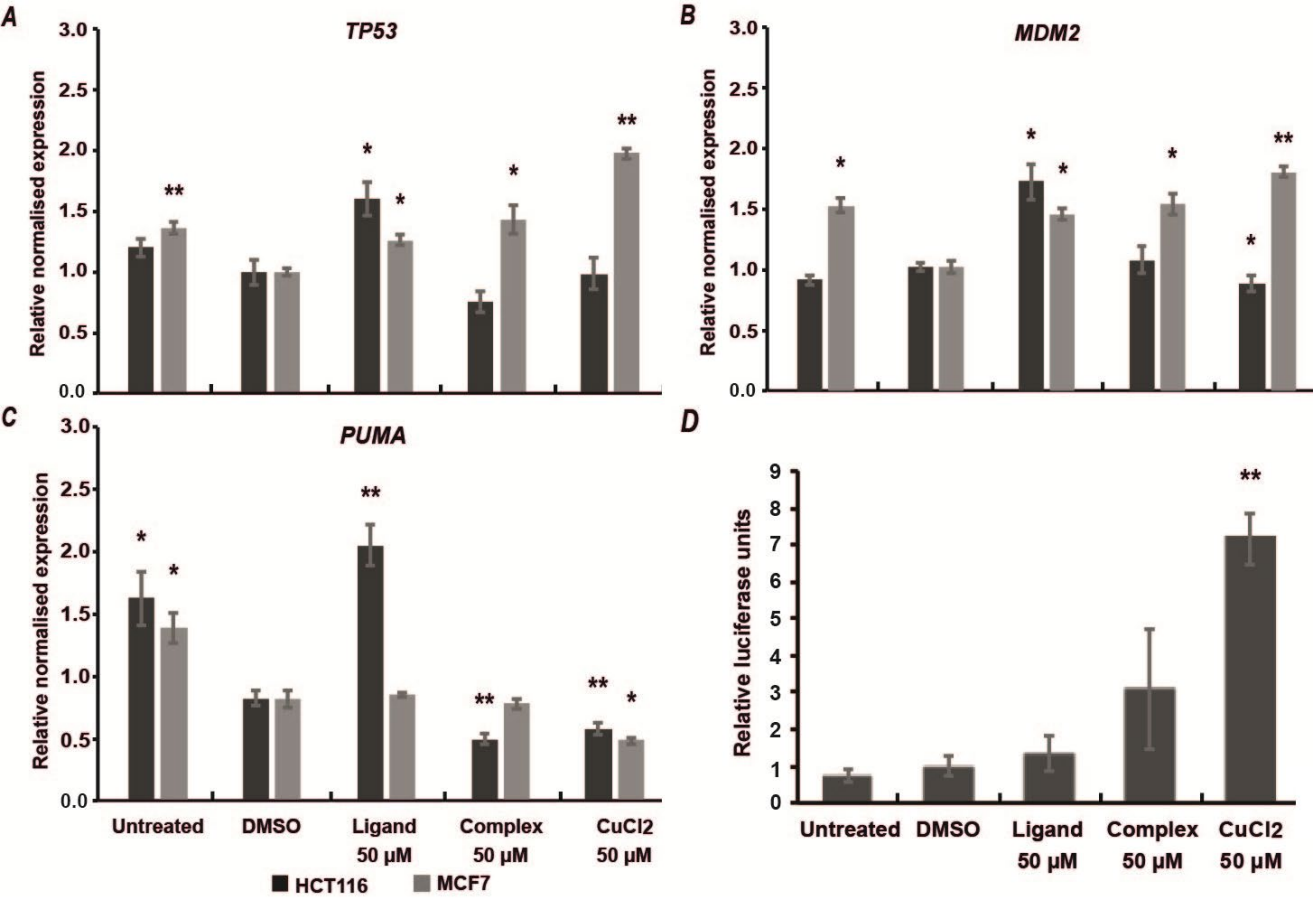
Complex and CuCl_2_ enhance p53 transcriptional functions.

Taqman real-time reverse transcription PCR (RT-PCR) with fluorescence-based detection system was used to quantitatively analyze gene expression. We noticed that in MCF7 cells Complex modestly enhanced expression of *TP53 *and *MDM2* genes while *PUMA* levels remained mostly unaffected (Fig. 3). In comparison, in HCT116 cells Complex slightly decreased *TP53* and *PUMA* expression levels and no effect on *MDM2*. Interestingly, Ligand increased expression of all three genes, especially *PUMA* in HCT116 cells (Fig. 3C). This can evidence the presence of early apoptosis since *PUMA* is a pro-apoptotic gene and its levels are regulated by p53 protein [35]. However, in MCF7 cells Ligand only moderately affected *TP53* and *MDM2*, and had no effect on *PUMA*. These results are in agreement with previous reports demonstrating that viability of tumor cells is more profoundly reduced by metal complexes compared to their respective free ligands [22]. In addition, we observed that CuCl_2_ enhanced expression of *TP53* and *MDM2* in MCF7 cells while showing almost no activity in HCT116 cells.

Relative normalized expression levels of *TP53* (A), *MDM2 *(B) and *PUMA* (C) genes in HCT116 and MCF7 cells after 24 h treatment with Ligand, Complex and CuCl_2_ at 50 µM. Data are expressed as mean ± S.D., n = 3; *p<0.05, **p<0.001. D – Complex is more potent than Ligand in luciferase-based p53 pathway activation assay. Although, CuCl_2_ demonstrates the most profound effect on p53 transcriptional activity (7-fold increase).

*PUMA* (aka *BBC3*) plays an important role in p53-regulated cell death via induction by DNA damage in a strictly p53-dependent manner. Activation of PUMA protein is a promising therapeutic approach aimed to inhibit tumor growth by triggering apoptosis [35]. As such, Ligand can be expected to have two potential mechanisms of action – via direct inhibition of MDM2, as previously reported [29], and through DNA damage like some other metal complexes [36, 37].

On our case, the difference in observed effect of Complex and Ligand in HCT116 and MCF7 might result from varying status of MDM2 expression in these cell lines. Apparently, overexpression of MDM2 in MCF7 cells alters cellular homeostasis and affects p53 activation.

To better understand the broader impact of Complex on p53-mediated transcriptional functions we performed additional quantitative assessment of signal transduction pathway activation in MCF7 cells. For that we used Cignal p53 Pathway Reporter Assay Kit (Qiagen) that constitutes a mixture of p53-inducible responsive construct (*Firefly* luciferase) and construct constitutively expressing *Renilla *luciferase for internal normalization.

The results suggest that Complex modestly enhanced p53-mediated Luciferase activity, although the signal does not have statistically significant difference from the controls (Fig. 3D). Meanwhile CuCl_2_ increased luciferase activity nearly 7-fold. This effect is in agreement with previously reported phenomena suggesting that transition metals (i.e. Cu^2+^, Zn^2+^) activate p53 and expression of its downstream targets [38]. The DNA binding interface of p53 contains Zn^2+^ ion that is required for proper folding and functioning [39]. Elevated concentrations of Cu^2+^ can partially displace Zn^2+^ from p53 binding site and in this way alter p53 transcriptional activity  [40].

**Figure 4:**
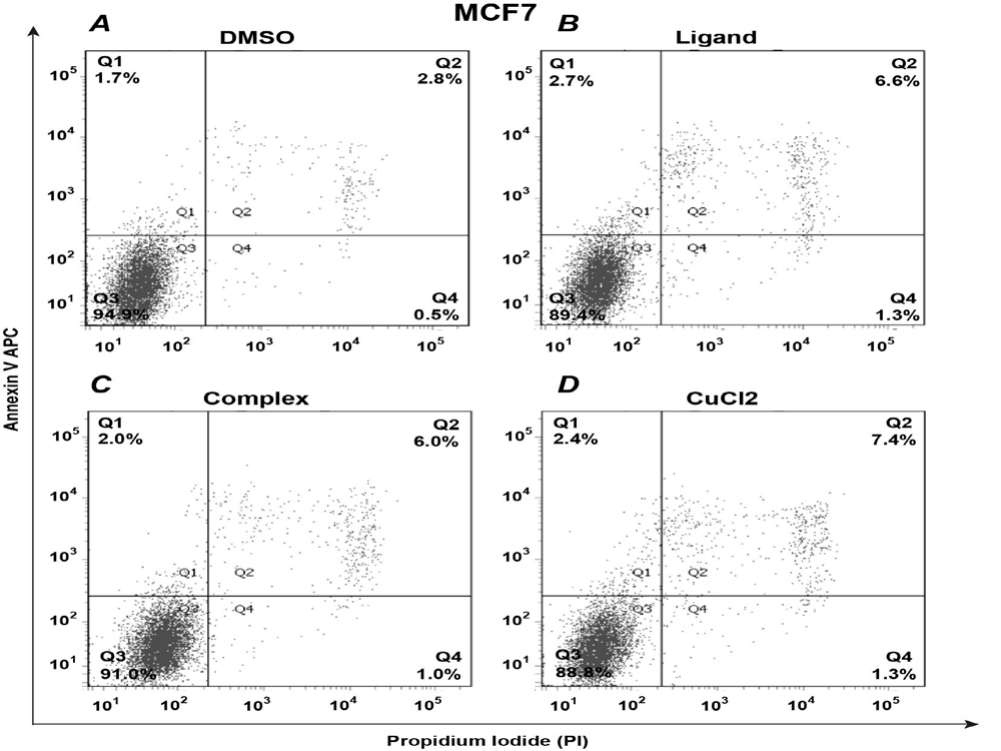
Isatin-Schiff base copper (II) complex induces apoptosis and necrosis in MCF7 cells.

To determine whether treatment with the compounds induces apoptosis MCF7 cells were treated with Complex (50 μM), Ligand (50 μM), CuCl_2_ (50 µM) and DMSO (1%, vehicle control) for 24 h. After that cells were stained with APC Annexin V and Propidium Iodide to evaluate percentage of apoptotic and necrotic cells by means of flow cytometry. Results indicated a minor increase in percentage of late apoptotic and necrotic cells upon treatment with Complex in comparison to DMSO control (Quadrants Q2 and Q4 in Fig. 4A and 4C). The results for cell treated with Complex and Ligand were almost identical. However, treatment with CuCl_2_ further increased the percentage of late apoptotic and necrotic cells (Quadrant Q2, Fig. 4D).

 MCF7 cells were treated with Complex (50 μM), Ligand (50 μM), CuCl2 (50 µM) and DMSO (1%, vehicle control) for 24 h, and then stained with APC Annexin V and Propidium Iodide to evaluate percentage of apoptotic and necrotic cells. Stained cells were analyzed by flow cytometry using BD FACSAria III and data processed with FlowJo software package. Quadrants represent following: Q1 – early apoptotic cells (Annexin V positive); Q2 – late apoptotic and necrotic cells (double positive); Q3 – healthy live cells (double negative); Q4 – necrotic cells (PI positive).

**Figure 5:**
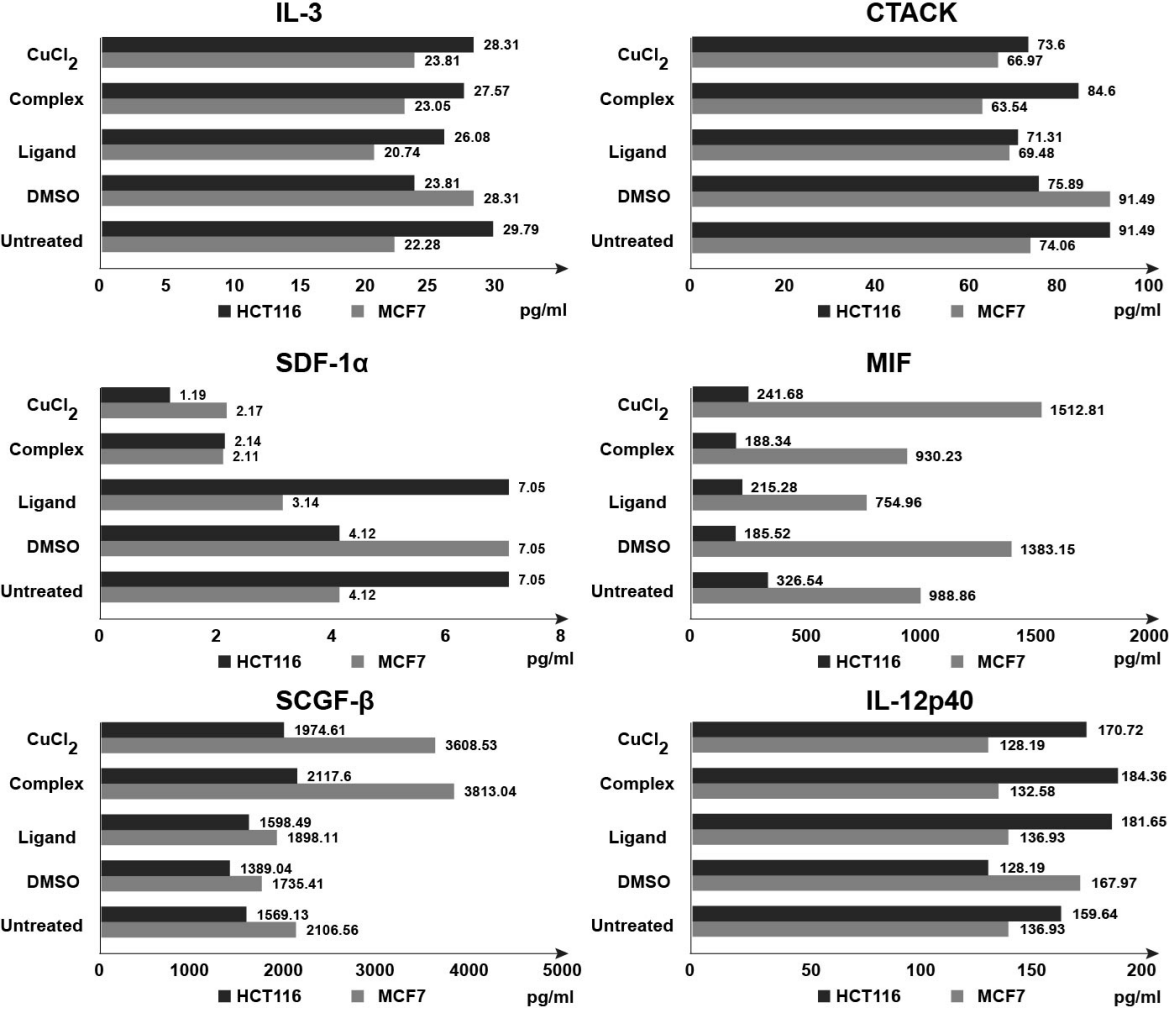
Complex and CuCl2 alter cytokine and chemokine secretion in tumor cells.

We determined the effect of Ligand and Complex on secretion of pro- and anti-inflammatory signaling molecules using Bio-Plex Pro Human Cytokine 21-Plex Immunoassay kit that allows simultaneous detection of a wide range of cytokines. To quantify the magnitude of secreted cytokines conditioned medium was collected after 24 h of treatment with Complex (50 μM), Ligand (50 μM), CuCl_2_ (50 µM) and DMSO (1%, vehicle control). We found that results for 6 out of 21 tested analytes revealed considerable alterations upon treatment with Complex. These analytes are inflammatory cytokines (IL-3, IL-12p40, MIF, SCGF-β) and chemokines (CTACK, SDF-1α) (Fig. 5). Interestingly, Complex demonstrated mostly opposite effect on the secretion of cytokines/chemokines in HCT116 and MCF7 cells. In HCT116 cells Complex increased levels of IL-3 1.2 times, CTACK –1.1 times, IL-12p40–1.4 times, SCGF-β – 1.5 times, decreased levels of SDF-1α – 2 times and did not affect MIF. On the contrary, in MCF7 cells IL-3 decreased 1.2 times, CTACK–1.4 times, IL-12p40–1.3 times, SDF-1α–3.3 times, MIF–1.5 times, and increased SCGF-β–2.2 times. Overall, these data suggest that Isatin-Schiff base copper (II) complex might be involved in p53-associated regulation of inflammatory processes. 

Conditioned medium from HCT116 and MCF7 cell lines harvested after 24 h treatment with Complex (50 μM), Ligand (50 μM), CuCl2 (50 µM), DMSO (1%, vehicle control) was subjected to multiplex immunoassay. Inflammatory cytokines (IL-3, IL-12p40, MIF, SCGF-β) and chemokines (CTACK, SDF-1α) showed notable alterations upon treatment with Complex and CuCl_2_. The results are presented as concentration of analytes (pg/ml) in supernatant.
